# More variety with the Journal of Cachexia, Sarcopenia and Muscle: JCSM Clinical Reports and JCSM Rapid Communications have both gone live

**DOI:** 10.1002/jcsm.12298

**Published:** 2018-04-02

**Authors:** Stefan D. Anker, Stephan von Haehling, Andrew J.S. Coats

**Affiliations:** ^1^ Division of Cardiology and Metabolism, Department of Cardiology (CVK); Berlin‐Brandenburg Center for Regenerative Therapies (BCRT); Deutsches Zentrum für Herz‐Kreislauf‐Forschung (DZHK) partner site Berlin; Charité Universitätsmedizin Berlin Berlin Germany; ^2^ Department of Cardiology and Pneumology University of Göttingen Medical Center Göttingen Germany; ^3^ IRCCS San Raffaele Pisana Rome Italy

**Keywords:** Journal, Cachexia, Sarcopenia, Muscle wasting, Frailty, JCSM Clinical Reports, JCSM Rapid Communications

Last year has brought more variety to the still too small publishing world of cachexia, sarcopenia, and frailty. Besides *JCSM Clinical Reports*,^1^ now our second open access daughter journal, named *JCSM Rapid Communications*, has gone live. We have two dedicated websites for these journals—until they become visible in big search databases like PubMed or Scopus, you can find all content of both journals at www.http://jcsm-clinical-reports.info and http://www.jcsm-rapid-communications.info/index.php/jcsm-rc. For those who love statistics, *JCSM Clinical Reports* so far has 3 editorials and 12 original papers and 1 review published, and *JCSM Rapid Communications*—having started only very recently—has 1 editorial and 2 original papers under its belt. As much as I would like to thank the whole editorial team in Göttingen and Berlin for their hard work in making these journal projects come alive, I would also like to thank the authors of these articles for entrusting us with their academic work and believing us thereby that we make these publications reputable and lasting homes of their work.

For the main *JCSM* journal, clinical trials,^2^, ^3^, ^4^, ^5^, ^6^ disease definition and regulatory issues,^7^, ^8^, ^9^, ^10^ epidemiological reports of broad interest,^11^, ^12^, ^13^ and significant basic science mechanistic insights^14^, ^15^, ^16^ as well as studies on pathophysiology questions^17^, ^18^, ^19^ will remain the main domain of what we aim to publish, but there are so many more things that deserve to be published and often only by a small margin don't make it into *JCSM*. We have a rejection rate for original reports that is >80%. For the best half of these papers, you will now find that we offer publication transfer to *JCSM Clinical Reports* or *JCSM Rapid Communications*, which naturally will have a higher acceptance rate, at least in the first few years. For *JCSM Clinical Reports*, this was about 50% in 2017. Papers that we accepted there included interesting reports on the importance of body mass index in chronic kidney disease^20^ and cancer,^21^ diagnostic^22^ and imaging studies^23^, and a report on exercise training in cancer patients^24^ or the social burden of weight loss in lung cancer.^25^ The first papers of *JCSM Rapid Communications* are now also online.^26^, ^27^ In the long run, we would think that *JCSM Clinical Reports* will be more clinically oriented as the title suggests and that *JCSM Rapid Communications* will be more oriented towards the basic science community of the wasting disorders field. We will all see whether it works out this way.

Both journals are open access journals. The world of publishing is full of such journals, and it is sometimes difficult to distinguish valid publication efforts from commercial publications with no academic aim mass published by the so‐called predatory publishers.^28^ Still, we believe it is the future of most journals where the aim is fast publication and free access for anyone and exclusion of the middle man of publishing (i.e. the publishers) making a healthy living of the distribution of knowledge (mostly funded by the public) to the public. Of course, there are limits to growth for open access journals—particularly in niche areas like ours. We simply have no marketing budget, and our publication—despite its leadership role in the field and good impact factor—is not available in most libraries of the world. Not even the best publisher in the world bundles an open access journal like ours with their general library offer. Also, many researchers favour submitting to what they call ‘normal’ print journals. For the average author that would mean that for publishing in our journal they would not need to pay the author publication charge from their all too limited research budget. Their organization as a whole would then of course pay through the library charge—assuming we make on to their rooster of journals made available to their faculty. We have heard colleagues ask whether such a future is possible for the *JCSM* mother journal. I don't know the answer to this yet, but a thought process is ongoing to this end. If you have thoughts on this, please let us know your opinion.

Stephan von Haehling is the editor‐in‐chief of both *JCSM Clinical Reports* and *JCSM Rapid Communications*. The work as co‐editor‐in‐chief for the *JCSM* mother journal has made him highly experienced about the field and the world of publishing—we look forward to his successful stewardship and cooperation in the *JCSM* journal family. Also the *JCSM Clinical Reports* and *JCSM Rapid Communications* open access journal will employ an ethical publishing standard similar to that outline for the main journal.^29^, ^30^ The journal is self‐published at this stage as Wiley did not want to take on the daughter journals yet. We are confident that this will change soon, and then the publication, editorial work, and readership experience will be fully the same for all three *JCSM* journals. On the way to success, we hope you join us contributing your work to this important field. Patients with cachexia, sarcopenia, and frailty deserve our work and success, and that requires exchange of information and ideas. We hope to provide with this growing body of journals a forum for this.

## Conflict of Interest

None declared.
